# Optimization of carbon footprint management model of electric power enterprises based on artificial intelligence

**DOI:** 10.1371/journal.pone.0316537

**Published:** 2025-01-03

**Authors:** Liangzheng Wu, Kaiman Li, Yan Huang, Zhengdong Wan, Jieren Tan

**Affiliations:** 1 Energy Development Research Institute, China Southern Power Grid, Guangzhou, China; 2 South China University of Technology, Guangzhou, China; 3 China Energy Engineering Group Guangdong Electric Power Design Institute Co., Ltd., Guangzhou, China; University of Mannheim: Universitat Mannheim, GERMANY

## Abstract

This study intends to optimize the carbon footprint management model of power enterprises through artificial intelligence (AI) technology to help the scientific formulation of carbon emission reduction strategies. Firstly, a carbon footprint calculation model based on big data and AI is established, and then machine learning algorithm is used to deeply mine the carbon emission data of power enterprises to identify the main influencing factors and emission reduction opportunities. Finally, the driver-state-response (DSR) model is used to evaluate the carbon audit of the power industry and comprehensively analyze the effect of carbon emission reduction. Taking China Electric Power Resources and Datang International Electric Power Company as examples, this study uses the comprehensive evaluation method of entropy weight- technique for order preference by similarity to ideal solution (TOPSIS). China Electric Power Resources Company has outstanding performance in promoting renewable energy, with its comprehensive evaluation index rising from 0.5458 in 2020 to 0.627 in 2022, while the evaluation index of Datang International Electric Power Company fluctuated and dropped to 0.421 in 2021. The research conclusion reveals the actual achievements and existing problems of power enterprises in energy saving and emission reduction, and provides reliable carbon information for the government, enterprises, and the public. The main innovation of this study lies in: using artificial intelligence technology to build a carbon footprint calculation model, combining with the data of International Energy Agency Carbon Dioxide (IEA CO_2_) emission database, and using machine learning algorithm to deeply mine the important factors in carbon emission data, thus putting forward a carbon audit evaluation system of power enterprises based on DSR model. This study not only fills the blank of carbon emission management methods in the power industry, but also provides a new perspective and basis for the government and enterprises to formulate carbon emission reduction strategies.

## Introduction

With the increasingly serious global climate change and China’s strategic goal of “peak carbon dioxide emissions, carbon neutral”, the power industry is gradually transforming from the traditional coal-fired power generation to a more diversified and clean direction. The accounting of carbon emission cost has become a key index to evaluate the green development of power enterprises. Although China’s power industry ranks among the top in the world in terms of installed capacity and power generation, there is still a lot of power generated by thermal power, especially coal-fired power. Therefore, optimizing the energy structure of the power industry and improving the management level of carbon emissions are the key tasks to deal with climate change. In the 21th century, one of the biggest issues facing human society is global climate change. The power sector is the primary source of carbon dioxide emissions worldwide, contributing around 40% of total emissions, according to research published by the International Energy Agency (IEA). For this reason, cutting carbon emissions in the electricity sector is essential to reaching global climate targets [1–3]. China has said unequivocally that “carbon dioxide emissions will reach the peak by 2030 and strive to achieve carbon neutrality by 2060” as the world’s top energy consumer and carbon emitter. The proposal of this goal is not only a solemn commitment to the international community, but also an inherent requirement to promote the transformation of China’s energy structure and high-quality development. Given this context, power enterprises’ control of their carbon footprints has emerged as a critical component in achieving the “double carbon” aim, since they are the primary source of energy production and delivery. However, the traditional carbon footprint management method has many shortcomings, such as incomplete data collection, low calculation accuracy and single emission reduction strategy. These problems seriously restrict the carbon emission reduction effect of power enterprises.

The green transformation and low-carbon development of the power industry have become the international consensus and a crucial direction for domestic policies, as it is one of the primary producers of carbon emissions. Building a thorough and efficient carbon audit evaluation system is especially crucial for conducting a methodical, scientific assessment of the power industry’s carbon emission status and impact on emission reduction. Currently, the power industy’s development exhibits the following broad features: First, the energy structure is gradually optimized, and the proportion of clean energy is gradually increased. Second, technological progress is accelerating, and the level of intelligence and digitalization is constantly improving. Third, the market demand is diversified, and emerging formats such as distributed energy and microgrid are developing rapidly. Fourth, the regulatory landscape is getting more stringent, and the industry’s ability to grow is increasingly being hampered by the need to reduce carbon emissions [4–6]. These attributes not only offer rich contextual data for the assessment of the power industry’s carbon audit, but they also present new specifications for the development of the evaluation system.

Artificial intelligence (AI) technology is developing at a rapid pace, and this has led to an expansion of its applications in carbon footprint management. Through big data processing, machine learning, deep learning, and other technological methods, AI can swiftly process and evaluate enormous amounts of carbon emission data, increasing the precision and effectiveness of carbon footprint calculations [7]. AI can also forecast the trend in carbon emissions going forward using both historical and current data, which is very helpful in developing scientific emission reduction plans. In the power industry, AI has been applied to many links, such as load forecasting, equipment management, information management, power market and so on. For example, load forecasting technology based on big data and machine learning can significantly improve the forecasting accuracy and provide important reference for power system scheduling and operation and maintenance. An intelligent inspection robot can increase the dependability and safety of equipment operation by enabling real-time monitoring and early defect detection [8, 9]. These applications provide powerful technical support for carbon footprint management of power enterprises.

With the increasingly serious problem of global climate change, governments and enterprises all over the world pay more and more attention to carbon emission management. However, the traditional carbon emission accounting methods and management models of power enterprises have shown many shortcomings. For example, Su, Yu [10] pointed out that the existing carbon emission management models often failed to make full use of the potential of big data and AI technology, which limited the accuracy of data processing and analysis. Zhao, Kou [11] thought that the existing research had obvious shortcomings in dealing with complex data and dynamic changes in the power industry, and often ignored the application of machine learning technology. In addition, Yang, Li [12] mentioned that the current carbon footprint management methods mainly relied on static data analysis and lacked effective response to real-time dynamic changes. Meanwhile, some studies failed to comprehensively consider the driving factors of carbon emissions, state evaluation, and corporate response measures [13, 14]. Fu and Zhou [15] demonstrated the irreplaceable role of comprehensive energy system as an important infrastructure in the rural revitalization strategy of China. Taking the actual energy system in northern rural areas as an example, they studied the collaborative optimization method of photovoltaic greenhouse and rural energy system, and revealed how to optimize and coordinate between agricultural production and meteorological sensitivity of photovoltaic power generation. The results showed that using this optimization method could significantly save energy consumption. Fu, Wei [16] discussed the importance of distributed energy planning and construction in meeting the requirements of modern agricultural electrification and cleaning, considered the impact of agricultural load control on carbon emissions, and proposed an agricultural load control model based on crop physiology and ecology. This model showed significant cost savings and carbon emission reduction benefits in the case of rural energy system in Hebei Province. Fu, Bai [17] emphasized the importance of agricultural microgrid in the process of modern agricultural production. Unlike traditional industrial microgrid, agricultural microgrid had unique characteristics on the load side, and its carbon emission was particularly important in the interaction between agriculture and energy. This study proposed a collaborative optimization method between greenhouse and microgrid, which verified its effectiveness in enhancing economic efficiency and low-carbon operation. This study also fully considered the impact of agricultural photosynthesis on energy carbon emissions, with a view to reducing energy carbon emissions through this cross-disciplinary collaborative optimization. This method not only provided a new perspective and methodological support for the practice of green production in power enterprises, but also drew lessons from the successful experience of agriculture and energy systems to achieve more significant carbon emission reduction effects in a wider range of application scenes.

In the existing carbon footprint management research, most models rely on traditional statistical methods and empirical formulas, but these methods have limitations in accurately reflecting the carbon emissions of power enterprises. For example, Li and Zhang [18] developed a carbon emission estimation model based on the traditional statistical regression method, but the results showed that this method could not fully consider the dynamic characteristics of energy structure and technological changes in complex power production environment. At the same time, Ridwana, Nassif [19] pointed out that the traditional linear regression model based on energy consumption data often underestimated the carbon emission reduction effect brought by the improvement of energy efficiency, resulting in inaccurate carbon footprint management. On the other hand, although the application of AI technology in carbon footprint management started late, some studies have preliminarily verified its potential advantages. Shi, Yang [20] applied the deep learning model to analyze the carbon emission data of power enterprises, which proved that this method had high accuracy and stability when dealing with large-scale nonlinear data. However, the current AI model still faces challenges in data quality, model complexity and universality in practical application. Especially when dealing with multi-source heterogeneous data and complex production environment, the existing AI model is still difficult to provide systematic solutions [21]. In addition, for the carbon footprint management model of power enterprises, the existing research mostly focuses on static or single-dimensional analysis, lacking a comprehensive and dynamic management framework. Li, Li [22] put forward an evaluation model based on carbon emission intensity of unit power production, but the model failed to consider the influence of energy structure diversity and production technology changes in power enterprises, so its applicability in low-carbon transformation was limited. In contrast, Zhao, Guo [23] tried to improve the accuracy of carbon emission estimation by combining life cycle analysis and energy efficiency assessment, but the research results showed that the flexibility and forecasting ability of the model still needed to be further improved in the changeable market and policy environment. The purpose of this study is to develop a scientific carbon footprint management model for power enterprises to address the aforementioned issues. Specifically, a more thorough and precise carbon footprint estimate methodology using big data and AI technologies is created. To find important influencing elements and potential for emission reduction, the carbon emission data of power companies is thoroughly mined and analyzed using machine learning algorithms. Applications of the drivers-state-response (DSR) model are made for the power industry’s carbon audit assessment. It comprehensively reflects the driving factors (such as energy structure, technological progress, policy environment, etc.), current state (such as carbon emissions, energy efficiency, etc.) and the industry’s response measures to carbon emission reduction (such as clean energy development, application of energy-saving and emission-reduction technologies, etc.) of the power industry to formulate scientific carbon.

The innovation of this study is that it combines big data and AI technology for the first time to establish an accurate carbon footprint calculation model, which can process large-scale data of power enterprises and conduct in-depth analysis, thus identifying the key factors affecting carbon emissions. At the same time, it uses machine learning algorithm to mine and evaluate carbon emission data, which is the first time in existing research. On this basis, the DSR model is introduced in this study to comprehensively evaluate the carbon audit of the power industry, which can effectively capture the driving factors, current situation and response measures of the industry in the process of carbon emission reduction. This comprehensive evaluation method is more comprehensive and in-depth than the previous single-dimensional analysis. In addition, it also applies the entropy weight—TOPSIS comprehensive evaluation method to the analysis of carbon emission reduction effect, which not only provides more scientific and reliable carbon information support, but also makes up for the shortcomings of the existing models in comprehensive evaluation. These innovations make this study have an important contribution to improve the scientific and practical aspects of carbon footprint management in power enterprises, and provide new ideas and methods for future related research.

The research of this study is of great significance. Firstly, it is an innovative promotion to the carbon footprint management model of power enterprises. The research aims to build a scientific and accurate carbon emission calculation model through AI technology, especially machine learning algorithm. This model can dig deeply into the key factors in the carbon emission data of power enterprises, and find out the significant factors affecting carbon emission and the opportunities for emission reduction, thus providing strong support for power enterprises to formulate effective carbon emission reduction strategies. Through the case analysis of two typical enterprises in China electric power industry-China Electric Power Resources and Datang International Electric Power Company, this study not only reveals the actual achievements of these enterprises in energy saving and emission reduction, but also reveals their advantages and disadvantages in coping with the challenge of carbon emission through data mining and evaluation methods. Secondly, the proposed carbon audit evaluation index system and its application have practical guiding significance for promoting the green development of power enterprises. By introducing authoritative data from IEA CO_2_ emission database and combining entropy weight -TOPSIS comprehensive evaluation method, this study provides a more systematic and scientific evaluation framework for carbon footprint management. This can not only help enterprises accurately grasp their carbon emission status, but also reveal the problems and shortcomings in the process of emission reduction, thus providing reliable carbon information support for the government, enterprises and the public and helping to formulate more scientific carbon emission reduction policies and measures. Finally, the discussion of carbon emission cost accounting method in this paper also has high application value. Under the background of global response to climate change and China’s strategic goal of “peak carbon dioxide emissions” and “carbon neutrality”, carbon emission cost accounting of power industry has become particularly important. This study analyzes the composition of carbon emission cost of power enterprises in detail, including carbon emission calculation, resource loss cost and environmental damage value, which provides scientific basis for enterprises to formulate green development strategies and investment decisions. In addition, the carbon audit evaluation method based on DSR model comprehensively considers the driving factors, state performance and response measures of power enterprises in the process of emission reduction, which provides a new idea for the improvement and optimization of carbon audit evaluation system. In a word, this study not only provides scientific evaluation tools and methods for carbon footprint management of power enterprises, but also has important practical significance for promoting the green development of power industry and achieving carbon emission reduction targets. Through systematic analysis and evaluation, this study provides strong support for the low-carbon transformation of the power industry, and provides valuable data basis for the formulation and implementation of relevant policies.

## Methods

### Present situation of China’s carbon emissions based on IEA CO2 Emissions Database

Since the industrial revolution, the power industry has experienced the development process from steam power generation to thermal power generation, and then to the diversified energy structure. The energy structure of the world’s power industry is currently undergoing a major transition, and renewable energy sources including hydropower, wind energy, and solar energy are progressively contributing to the production of electricity. China’s power sector has also accomplished a great deal in recent years. Not only does it have some of the highest installed power capacity and power generation in the world, but it has also made significant strides toward the development of clean energy. In terms of the structure of power production, thermal power generation dominates China’s power sector. Nevertheless, the share of clean energy power generation is growing annually. Gas-fired power generation and coal-fired power generation make up the bulk of thermal power generation, with coal-fired power generation holding a leading position. Nonetheless, the share of renewable energy, such as solar and wind power, in power generation has grown annually due to the ongoing advancements in clean energy technology and cost reductions. Nuclear power is a clean, effective energy source that contributes significantly to China’s electricity output.

**Fig 1:**
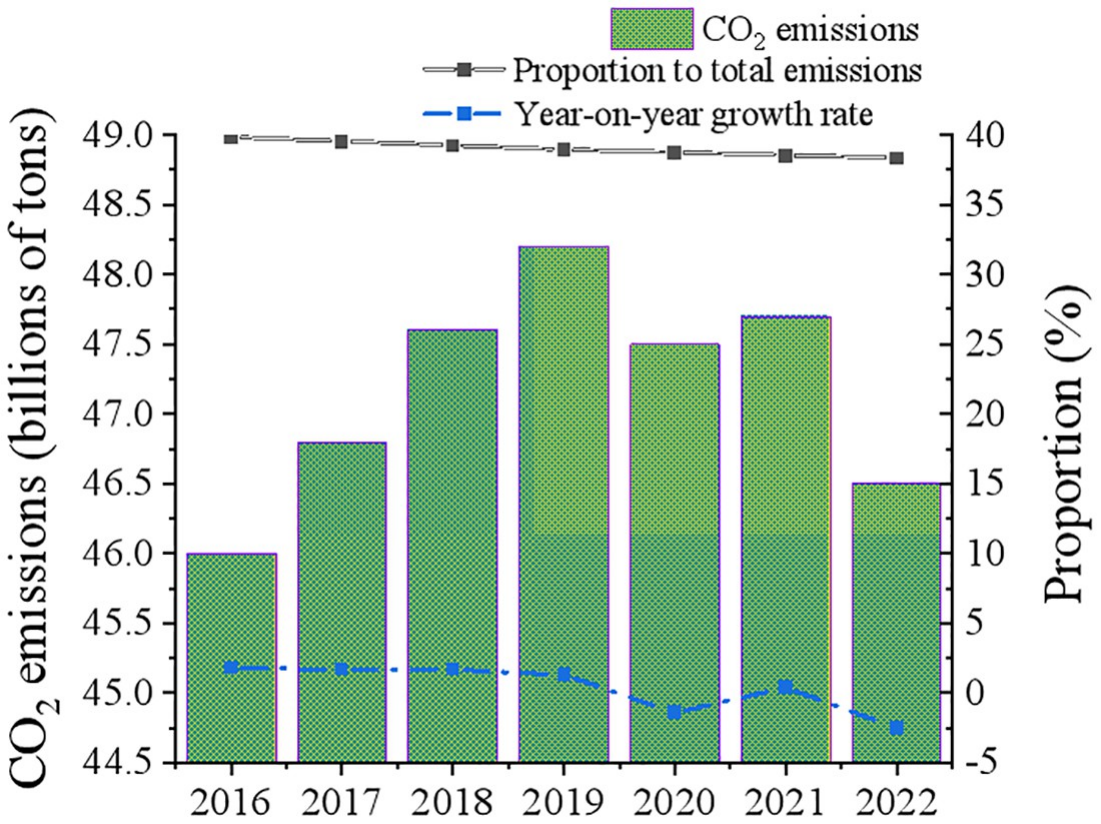
Overview of carbon emissions of power industry in China from 2016 to 2022.

With China’s economy expanding quickly and the country’s citizens enjoying higher living standards, there is an increasing need for power consumption. The primary sectors of electricity use are industrial, commercial, and residential, with industrial electricity making up a sizable share of all three. The rapidly evolving fields of electric vehicles and smart houses are causing a progressive shift in the structure of power consumption as well [24–26]. This study makes use of the IEA CO_2_ Emissions Database to more precisely analyze the current state of carbon emissions in China’s power industry. The database offers comprehensive information on carbon dioxide emissions from both domestic and international energy operations, which is very helpful in assessing the carbon emissions of the power sector. China’s power industry’s carbon emissions from 2016 to 2022 are displayed in Fig 1. It demonstrates that China’s power industry’s carbon emissions have been trending toward first rising and then falling in recent years. Between 2016 and 2019, the power industry’s carbon emissions increased, but at a slower rate due to changes in the energy structure and the rise in power demand. 2020 saw a decrease in the demand for power due to the global COVID-19 pandemic and changes to the domestic economic structure, which led to a negative increase in carbon emissions. In 2021, with the economic recovery, carbon emissions rebounded slightly, but the growth rate slowed down significantly. By 2022, carbon emissions will decline again, which shows that China’s power industry has achieved positive results in emission reduction.

In China’s power industry, thermal power generation—particularly coal-fired power generation—is the primary source of carbon emissions. As a result, energy structure optimization is crucial for lowering carbon emissions in the power sector. China has successfully decreased the power industry’s carbon emission intensity in recent years by accelerating the development of clean energy and encouraging the conversion of coal-fired power to ultra-low emission sources. Regional variations in the power industry’s carbon emissions are evident. The eastern region’s strong demand for electricity and established economy have resulted in comparatively high carbon emissions. According to the research of Xie, Wang [27], due to the developed economy and dense population, the eastern coastal areas had relatively high-power demand and relatively large carbon emissions. The power consumption of eastern provinces such as Jiangsu and Guangdong have been in the forefront of the country for a long time, which was closely related to its economic development level and industrialization level. In the research of Zhu, Wang [28], it was considered that the proportion of thermoelectric production in the eastern region increases due to the increase of heating demand in winter, which led to the corresponding increase of carbon emission level. Comparatively speaking, Zheng, Deng [29] considered that the western region’s carbon emission level was kept at a low level due to the relatively backward economic development, low total electricity demand and abundant clean energy resources. In western provinces such as Qinghai and Yunnan, the seasonal fluctuation of power demand had little impact on the overall carbon emissions. In these areas, due to the abundant solar and wind energy resources in summer, they can better cope with the increase in power demand while maintaining low-carbon emissions. Therefore, the power structure and carbon emission characteristics in the western region were in sharp contrast with those in the eastern region.

### Carbon emission cost accounting of electric power enterprises

The power industry is progressively shifting away from the conventional method of coal-fired power generation and toward a more diverse and clean direction due to the severity of climate change on a worldwide scale and China’s strategic aim of “peak carbon dioxide emissions and carbon neutrality”. The accounting of carbon emission costs has developed into a crucial metric in this process for assessing the degree of green development of power companies. In terms of installed capacity and power generation, China’s power industry is among the best in the world. Nonetheless, a significant amount of power is still generated by thermal sources, particularly coal-fired power. The power industry’s energy structure is gradually being optimized due to the rapid development of renewable energy sources like solar and wind power [30, 31].

The accounting content of carbon emission cost of power enterprises covers many aspects, the first is the accurate calculation of carbon emission. This requires enterprises to use scientific calculation methods to estimate carbon emissions based on data such as power generation and coal consumption. Other greenhouse gas emissions should also be considered to fully reflect the carbon emissions in the process of power production. In addition to carbon emissions, resource loss cost is also an important part of carbon emission cost accounting. This includes the cost of procurement, transportation and storage of fossil energy such as coal. In the accounting process, it is necessary to combine resource loss with carbon emissions to reflect the resource consumption cost caused by carbon emissions. The value of environmental damage is also a part that cannot be ignored. Although this part of the cost is difficult to measure directly, it can be estimated by introducing an environmental damage assessment model. It represents the value that electric power enterprises should bear for the potential damage to the environment caused by greenhouse gas emissions.

**Fig 2:**
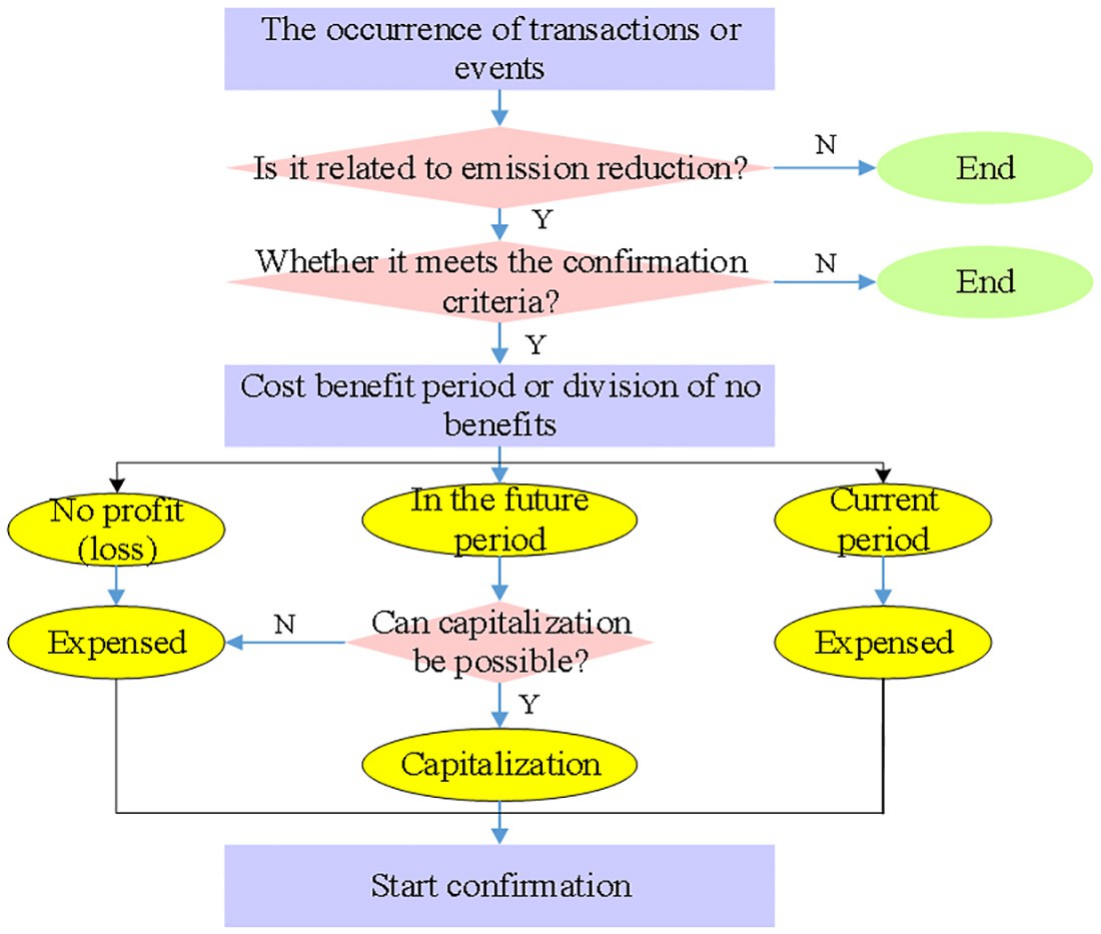
Specific confirmation process of carbon emission cost.

To maintain the accuracy and scientific nature of accounting, a set of guidelines should be adhered to when verifying the carbon emission cost of power enterprises. The definability principle requires that the carbon emission cost must be clearly defined and accurately understood and applied by enterprises. The principle of measurability emphasizes that the cost of carbon emissions should be accurately measured in monetary units and reflected in the accounting statements of enterprises. In order for the carbon emission cost to accurately reflect the carbon emissions during the power production process, it must be closely tied to the production and operation activities of businesses, according to the principle of correlation. Fig 2 illustrates the specific confirmation process of carbon emission cost.

In the specific accounting process, firstly, it is necessary to collect and sort out relevant data such as power generation, coal consumption and investment in environmental protection facilities of electric power enterprises. Then, carbon emissions are calculated according to these data and scientific calculation methods [32–34]. Then, carbon emissions, resource loss cost, environmental damage value, carbon emission reduction cost and carbon transaction cost are included in the accounting scope for comprehensive calculation. Finally, the accounting results are reflected in the accounting statements of enterprises, which provides strong support for the decision-making and green development of enterprises.

### Data mining of enterprise carbon emissions based on machine learning

The process of using algorithms to find hidden information in huge datasets is known as data mining. This procedure, which is typically associated with computer science, is carried out using a variety of techniques, including pattern recognition, machine learning, statistics, online analytical processing, information retrieval, and expert systems. Pretreatment, integration, selection, and preparation of data are all steps in the data mining process. Its goal is to find knowledge that users are interested in, which is acceptable, understandable and applicable, and supports specific applications to find problems. There are many algorithms in data mining, including classification, regression analysis, clustering, association rules and so on.

This study uses machine learning algorithm to deeply mine the carbon emission data of power enterprises, and choose decision tree algorithm as the main tool. This algorithm is superior in dealing with regression and classification problems, and has outstanding ability in dealing with data characteristics and prediction results. Microsoft decision tree algorithm is suitable for complex classification and regression problems, and its obvious advantages are that the model is easy to explain, the results are intuitive, and it can effectively deal with nonlinear data relationships, so this algorithm can meet the complexity requirements of carbon emission data. In the data preprocessing stage, this study first collects the original data from enterprise database and official website from 2020 to 2022, including power generation, fuel consumption and investment in environmental protection facilities. Then, it cleans the data, removes the missing values and abnormal values, and fills the missing data and correct the abnormal values through mean interpolation and interpolation method. On this basis, it also unifies the data format to ensure the consistency of data from different sources to facilitate the subsequent analysis, and standardized the data, and normalized all numerical data to values in the interval of [0, 1], thus eliminating the influence brought by different dimensions. In the feature extraction process, the importance of features is evaluated by decision tree algorithm, and it selects the features that have a significant impact on carbon emissions, such as total electricity production, coal consumption and environmental protection investment. At the same time, according to the business requirements and model requirements, it has constructed some new features, such as carbon emission intensity and clean energy ratio, which are helpful to improve the prediction performance of the model. Subsequently, in the model training step, it uses the decision tree algorithm to build the model. This algorithm creates a decision tree by dividing the data set into multiple subsets, in which each node represents the test conditions of feature attributes and the branches represent the results of feature attributes. In order to prevent the model from over-fitting, it sets parameters such as the maximum depth of the tree and the minimum number of sample splits, and uses cross-validation method to evaluate the generalization ability of the model to optimize the model parameters. Finally, the model is verified by training set and test set to ensure its accuracy and stability.

In this study, decision tree algorithm, Transformer model and entropy weight -TOPSIS method are selected, which are based on their unique advantages in processing carbon emission data and comprehensive evaluation. Decision tree algorithm is a kind of supervised learning technology, which has clear decision rules and is easy to explain. It can effectively handle large-scale datasets and provide an intuitive understanding of the importance of variables. Microsoft decision tree algorithm is chosen because it is excellent in dealing with the prediction modeling of discrete and continuous attributes, can accurately divide the data feature space, and is helpful to identify the key factors affecting carbon emissions. Transformer model is an important progress in the field of natural language processing in recent years, which can deal with complex sequence data. Because of the need to deal with a large number of time-series carbon emission data, Transformer model can capture long-distance dependence and effectively deal with unstructured information in the data. Compared with the traditional time series analysis method, the Transformer model is outstanding in dealing with large-scale data and provides more accurate analysis results. Entropy weight -TOPSIS method is a comprehensive evaluation method, which combines the advantages of entropy weight method and TOPSIS method Entropy weight method can objectively determine the weight of each evaluation index, while TOPSIS method ranks the schemes by comparing the distance between them and the ideal scheme. This method can comprehensively consider multiple evaluation indexes and provide scientific evaluation results through quantitative analysis. Compared with other evaluation methods, entropy weight -TOPSIS method has strong comprehensive analysis ability and accuracy when dealing with multi-dimensional and multi-objective evaluation problems. Table 1 shows the comparative analysis:

**Table 1:** Method comparative analysis.

Method	Advantages	Disadvantages
Decision tree algorithm	Easy to implement and explain; Suitable for processing large-scale datasets; Provide an intuitive understanding of the importance of variables.	Sensitive to noise data; May be over-fitted; For complex problems, deeper trees may be needed.
Support vector machine (SVM)	Has strong classification ability in high-dimensional data processing.	The process of model training and optimization is complex; High requirements for computing resources; It is difficult to process large-scale data.
Transformer model	Able to process complex sequence data; Effectively capture long-distance dependencies; Excellent performance in handling large-scale data.	High demand for computing resources; Model training time is long; May be too complex for a small dataset.
Recurrent neural network (RNN)	Has advantages in time series data processing; Suitable for processing sequential data.	Gradient disappearance is easy to occur in long sequence data processing; Training time is longer.
Entropy weight -TOPSIS method	The objective weight calculation and the distance comparison of ideal schemes are combined. Suitable for multi-dimensional and multi-objective evaluation problems.	Need accurate data preprocessing; For data without clear objectives, the evaluation may not be effective enough.
Weighted average method	Simple to deal with multi-index comprehensive evaluation; Easy to implement.	Depend on the weight set subjectively; It may not fully reflect the actual importance of evaluation indicators.

The decision tree method is a supervised learning technique used in data mining for problems with regression and classification. It divides the dataset into smaller groups iteratively to construct a decision tree. When building a decision tree, each node stands for the feature attribute’s test condition, and each branch represents the feature attribute’s result within a given value range. A category or a certain number is stored in each leaf node. The Microsoft SQL Server Analysis Services offers the Microsoft decision tree method, a classification and regression technique that is particularly well-suited for predictive modeling of discrete and continuous attributes. By constructing a decision tree model, the algorithm divides the feature space of data instances into several disjoint subsets, and each subset corresponds to a category or regression value.

**Fig 3:**
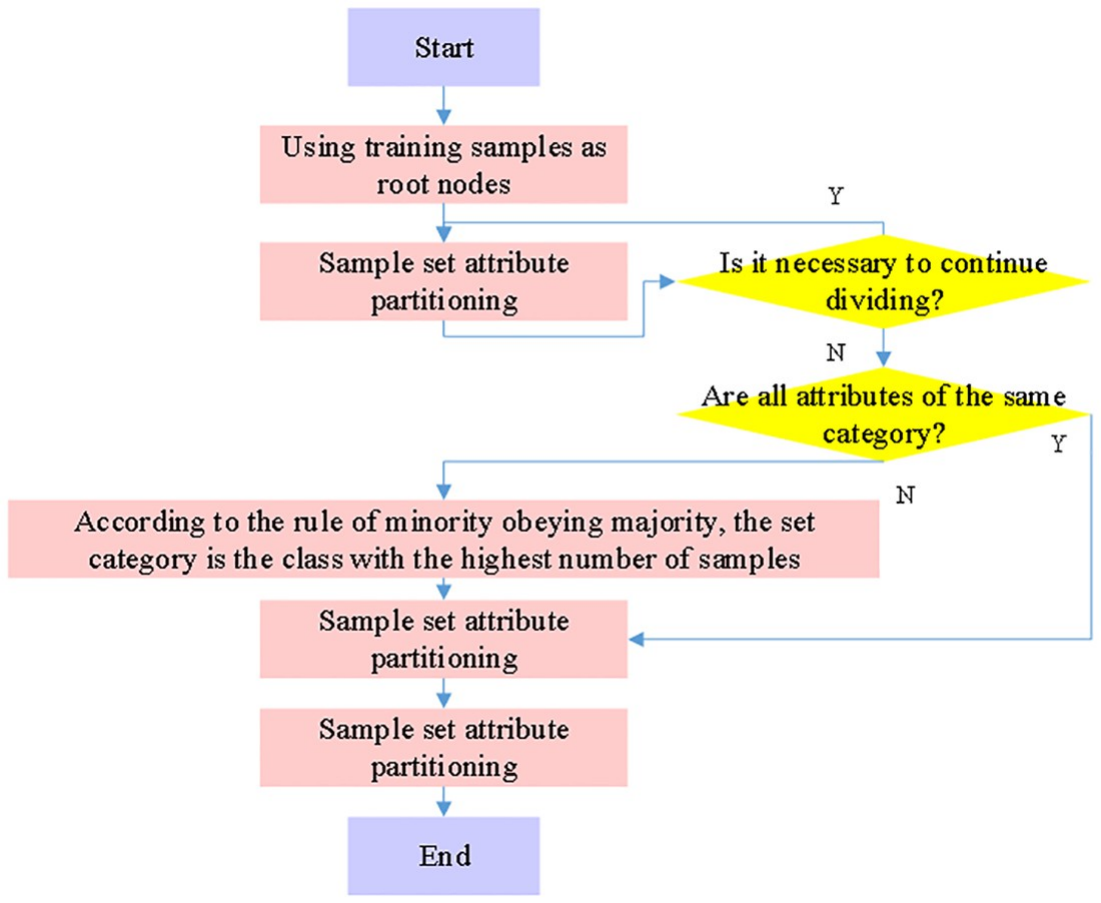
Decision tree construction process in Microsoft decision tree algorithm.

The Microsoft decision tree algorithm meticulously plans a number of breaks in the tree structure to create an effective data mining model. The method adds a new node to the model whenever it finds a substantial correlation between the input data’s columns and the predictable columns. “Feature selection” is a technique used by the Microsoft decision tree algorithm to help identify the most beneficial qualities. By preventing unnecessary characteristics from taking up processor time, all of these strategies contribute to increased performance and higher-quality analysis. The decision tree creation procedure is depicted in Fig 3. In order to assist users in extracting valuable knowledge and information from massive datasets, this technique is mostly utilized in the domains of data mining and machine learning.

Prior to engaging in carbon emission data mining, businesses’ carbon emission-related data must be gathered and sorted. Power generation, fuel consumption, environmental protection facility operation data, and production process parameters are a few examples of these data [35, 36]. Preprocessing the data is required to guarantee the precision and efficacy of data mining. This includes data cleansing, unifying the format, handling outliers, and other procedures. Preparing the data is a crucial first step in the data mining process. This covers tasks like feature extraction, data conversion, and cleaning. Transformer architecture can handle unstructured data, including text data. Transformer architecture, a significant advancement in natural language processing in recent years, offers a fresh viewpoint on evaluating English instruction since it can manage complicated text material and identify long-term relationships in sequences. In enterprise carbon emission data mining, a large number of carbon emission data need to be collected and processed first. These data may include energy consumption, production process, equipment status and other information. Because the Transformer model can process the sequence data, it is necessary to properly preprocess these data, such as time series transformation and normalization, so that the model can be better understood and processed.

Transformer architecture is the foundation for the construction of a multi-layer encoder-decoder model. The input text data must be transformed by the encoder into a set of feature vectors, which the decoder then uses to produce the evaluation results of the students’ learning. In order to maximize the model’s performance, the parameters and structure are modified during the model-building process based on the unique requirements. The Transformer model, which is utilized to describe the dependency between any two points in the sequence, is based on a self-attention process. The following is the equation used to calculate the attention weight:

$$Attention(Q,K,V)=softmax(\frac{QK^{T}}{\sqrt{d_{k}}})V$$ (1)

*Q* is the query matrix. *K* is the key matrix. *V* is the value matrix, and *d*_*k*_ is the dimension of the key vector, which is used to scale the dot product to avoid gradient disappearance or gradient explosion.

### Carbon audit evaluation of power industry based on DSR model

In the power industry, the management of carbon emission cost is a multi-dimensional and whole-process systematic project, and the selection of its indicators must take into account the comprehensive consideration before, during and after. First, considering pre-control, the choice of carbon emission cost indicators should be made with the low-carbon development strategy of the business in mind. This will guarantee that the indicators chosen can effectively steer the business in the direction of a low-carbon future. This requires enterprises to consider not only economic benefits, but also the carbon emission cost of projects when formulating investment strategies, and give priority to those emission reduction technologies or projects with significant low-carbon benefits and high cost-benefit ratio. Meanwhile, enterprises need to pay close attention to national and local laws and regulations on carbon emissions to ensure that the selected indicators meet the policy requirements and avoid compliance risks.

In the process control stage, real-time monitoring and dynamic adjustment of carbon emission cost is very important. Enterprises need to establish a perfect carbon emission monitoring system and use advanced information technology to monitor carbon emissions in real time and accurately. On this basis, enterprises should also regularly carry out cost-benefit analysis, compare the actual effect of emission reduction measures with the expected goals, and adjust emission reduction strategies in time to ensure effective control of emission reduction costs [37, 38]. In addition, enterprises need to strengthen risk management, identify and evaluate potential risks related to carbon emission costs, such as policy changes and market price fluctuations, and formulate effective countermeasures to ensure the stable operation of enterprises.

In the post-event control stage, the performance evaluation and continuous improvement of carbon emission cost is an indispensable part. Enterprises should establish a scientific performance evaluation system to quantitatively evaluate the effect of emission reduction, and take the evaluation results as the basis for improvement. Through the feedback mechanism, enterprises can timely understand the effectiveness and shortcomings of emission reduction work, and provide direction for subsequent improvement. At the same time, enterprises should actively fulfill their social responsibilities, compile and publish social responsibility reports and mass satisfaction questionnaires, disclose carbon emission cost information to the public, and enhance the transparency and social responsibility of enterprises. This will not only help to enhance the brand image of enterprises, but also set a low-carbon development model for other enterprises. Table 2 shows the summary of the three dimensions: before, during and after.

**Table 2:** Three control dimensions: Before, during and after.

Quasi hierarchy	Index layer
Prior control	Environmental protection equipment purchase fee
	Low carbon R&D cost ratio
	Identify carbon emission source expenditure
	Low-carbon training cost ratio
	Expenditure related to environmental education
In-process control	Carbon emission monitoring fee
	Maintenance fee of environmental protection facilities
	Cost ratio of carbon emission reduction governance
	Excessive carbon emission penalty
	Expenditure on technological innovation
Feedback control	Carbon emission loss cost rate
	Developing low-carbon new customer expenditure
	Cost of mass satisfaction survey
	Project system implementation cost

**Fig 4:**
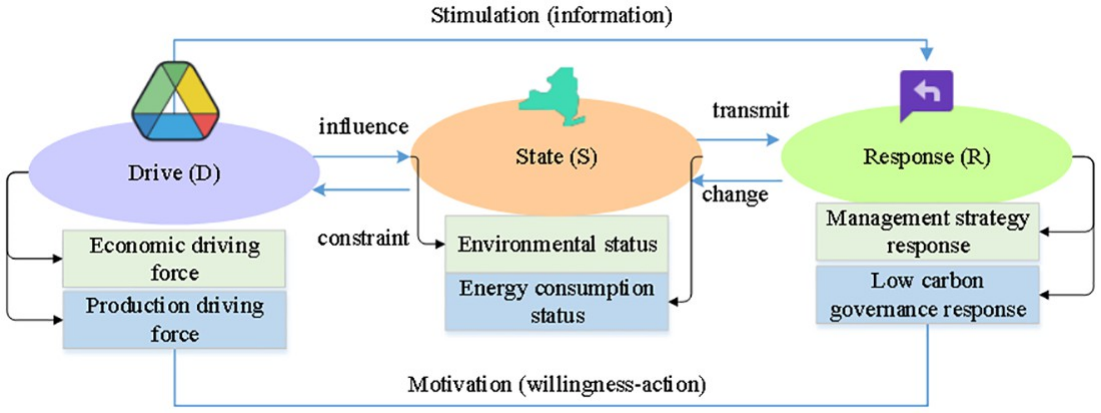
DSR model diagram.

DSR model is put forward by the United Nations Commission on Sustainable Development, which aims to understand and evaluate the impact of social and economic development on the natural environment through three key dimensions (drivers, state and response), as well as human responses and countermeasures to these impacts. In the DSR model, the drivers usually refer to the potential causes or forces that lead to environmental changes in the socio-economic system. The state refers to the state or characteristics of the system at a certain point in time, reflecting the results of the system affected by the drivers. Response usually refers to the policies, technologies and management measures taken by the government, enterprises, the public and other subjects to cope with environmental changes. The DSR model diagram is shown in Fig 4.

DSR model aims to understand and evaluate the impact of social and economic development on the natural environment through three key dimensions-Drivers, State and response, as well as the human response and response measures to these impacts. In the DSR model, the driving factors refer to the potential causes or forces that cause environmental changes. In the power industry, these factors usually include the adjustment of energy structure, technological progress and policy environment. For example, the transition from fossil fuel to renewable energy and the application of carbon capture and storage technology are all important driving factors. By analyzing these factors, we can identify the main forces that affect carbon emissions and formulate targeted carbon emission reduction measures. In China, the support of national policies for the development of clean energy is regarded as a key driver for the transformation of the power industry. State represents the state or characteristics of the system at a specific point in time, reflecting the results of the system being affected by driving factors. In carbon audit evaluation, status mainly refers to the carbon emission status and energy efficiency of enterprises, such as carbon dioxide, sulfur dioxide and soot emissions, coal consumption rate of power supply and comprehensive auxiliary power consumption rate of thermal power business. These state indicators can effectively reflect the carbon emission level and energy utilization of enterprises at different time points. Response refers to the policies, technologies and management measures taken by the government, enterprises and the public to cope with environmental changes. In the power industry, these response measures include the development and application of clean energy, the implementation of carbon capture and storage technology, ultra-low emission transformation and environmental protection investment. Through the analysis of these response measures, people can evaluate the positive actions of enterprises in reducing carbon emissions and improving energy efficiency and their effects. When applying DSR model to carbon audit evaluation of power industry, firstly, identify and quantify the driving factors of power enterprises, such as economic indicators such as total assets, total profits and power sales, as well as production indicators such as power generation and coal consumption. Then, according to the analysis results of driving factors, the carbon emission status of enterprises is evaluated, and environmental performance and energy consumption are concerned to judge the current situation of enterprises in carbon emission reduction and energy efficiency improvement. Finally, it analyzes the response measures taken by enterprises in dealing with carbon emissions, and evaluates their effectiveness, including management strategies and related indicators of low-carbon governance.

Through the above steps, DSR model can systematically reveal the changing mechanism of carbon emission in power industry under the action of external driving factors, and how all parties can promote the adoption of positive measures through feedback mechanism after the carbon emission status changes, and further promote the development of the whole industry in the direction of low carbon and environmental protection through virtuous circle mechanism. Based on the actual cases of two electric power enterprises in China, this study constructs a carbon audit evaluation index system with 18 indexes, and analyzes it by using entropy weight -TOPSIS comprehensive evaluation method. This process not only shows the practical application of DSR model in carbon audit evaluation, but also provides scientific basis and optimization direction for carbon emission reduction strategy of power enterprises.

Based on the existing DSR model, time series analysis method is introduced to capture the dynamic changes of carbon emissions in power industry. Time series analysis is to model and predict the regularity of time series data, thus reflecting the trend and periodic changes of data over time. In this study, a time series regression model (such as ARIMA model) is used to analyze the carbon emission data of power enterprises from 2020 to 2022 to identify the dynamic changes and key driving factors of carbon emissions at different time points. The time series analysis results of carbon emission data of China Resources Power and Datang International Power Generation Co., Ltd. from 2020 to 2022 is shown in Table 3. According to the time series analysis results in Table 3, it shows that the comprehensive evaluation index of China Resources Power presents an increasing trend year by year, from 0.5458 in 2020 to 0.6270 in 2022. This shows that the company has achieved remarkable results in promoting low-carbon energy and reducing the proportion of thermal power. The increase of driving factor D shows that the driving force of economy and policy is gradually enhanced, the promotion of state factor S shows the continuous improvement of carbon emission control, and the steady growth of response factor R reflects the continuous investment and optimization of low-carbon governance measures. In contrast, the comprehensive evaluation index of Datang International Power Generation Co., Ltd. fluctuates greatly, especially in 2021, which is related to the company’s retrogression in sulfur dioxide and carbon dioxide emission reduction. However, the company’s index rebounded in 2022, indicating that its carbon emission management effect has improved after adjusting its management strategy and strengthening investment in environmental protection. This result further proves the importance of time series analysis in capturing the dynamic changes of carbon emission management.

**Table 3:** Time series analysis results of carbon emission data of China Resources Power and Datang International Power Generation Co., Ltd. from 2020 to 2022.

Year	Enterprise	Drivers D	State S	Response R	Comprehensive evaluation index
2020	China Huarun Electric Power	0.65	0.55	0.45	0.5458
2021	China Huarun Electric Power	0.68	0.60	0.50	0.5800
2022	China Huarun Electric Power	0.70	0.65	0.55	0.6270
2020	Datang international power generation	0.60	0.50	0.30	0.5333
2021	Datang international power generation	0.62	0.55	0.20	0.4658
2022	Datang international power generation	0.64	0.60	0.35	0.5467

D-S mechanism reveals the internal logic of how the carbon emission state of power industry changes under the action of external drivers. The S-R mechanism reflects the process of how to urge all parties to take active measures through the feedback mechanism after the carbon emission status changes in the power industry. The R-D mechanism shows how the power industry can continuously generate new drivers through the virtuous circle mechanism after the implementation of the response measures to promote the development of the whole industry in the direction of low carbon and environmental protection. Based on the cycle mechanism of DSR model, combined with the characteristics of power industry and the actual demand of carbon reduction and emission reduction, an evaluation index system with 18 indicators is constructed to comprehensively evaluate the carbon reduction and emission reduction of power enterprises, as shown in Table 4.

**Table 4:** Carbon audit evaluation indicators based on DSR model.

First index	Second index	Third index
Drivers D	Economic D1	Total assets D11
		Total profit D12
		Electricity sales D13
	Sound field D2	Power generation D21
		Heat supply D22
		Coal consumption D23
State S	Environment S1	Smoke emission performance S11
		SO2 emission performance S12
		CO2 emission performance S13
	Energy consumption S2	Power supply coal consumption S21
		Comprehensive auxiliary power consumption rate of thermal power business S22
Response R	Management strategy R1	Total soot emission decreased R11
		Total SO2 emission reduction R12
		Total CO2 emission reduction R13
	Low-carbon governance R2	Ultra-low emission units accounted for R21.
		Wind power installed capacity R22
		Solar power generation installed capacity R23
		Environmental protection investment R23

Data mining is a process of discovering hidden information in large datasets through algorithms, which involves many technologies such as pattern recognition, machine learning and statistical analysis to support problem discovery in specific applications. In this study, machine learning algorithms such as decision tree method, regression analysis and cluster analysis are selected to mine and analyze the carbon emission data of power enterprises. Specifically, decision tree algorithm, as a supervised learning technology, is used to deal with classification and regression problems. Microsoft decision tree method provided by Microsoft SQL Server Analysis Services is used, which is suitable for forecasting and modeling of discrete and continuous attributes. When constructing the decision tree model, each node represents the test conditions of feature attributes, and each branch corresponds to the results within the range of feature attribute values. According to the characteristics of the dataset, the depth of the tree is adjusted (the maximum is set to 15), and the minimum sample number of leaf nodes is set to 5 to balance the complexity and accuracy of the model. At the same time, Gini index is used as the classification standard to optimize the tree construction process. In order to prevent over-fitting, pruning technology is applied and the minimum information gain threshold (0.01) is set.

In the construction process, the features that contribute the most to the prediction results are screened by calculating the importance of features, and the performance of the model is verified by using the 10-fold cross-validation method, thus improving the robustness and accuracy of the model. In data preprocessing, firstly, data cleaning, as the first step of preprocessing, aims to remove noise and inconsistent data in the dataset. Through statistical analysis and visualization methods, abnormal values and unreasonable data are identified. For example, for abnormal data such as fuel consumption or power generation, IQR method is used to detect and eliminate outliers, and repeated detection algorithm is used to eliminate possible data redundancy. Secondly, the treatment of missing values is also very important, because its existence may affect the accuracy and robustness of the model. The missing values of the data are analyzed, and corresponding processing methods are adopted according to the specific conditions of different characteristics. For key features (such as energy consumption data), interpolation method is used to fill in missing values to ensure the continuity and accuracy of data. For a small number of missing non-critical features (such as some equipment status data), the mean filling method is adopted. In addition, for features with high missing ratio, people choose to delete these features directly to avoid introducing too much uncertainty. Finally, after completing data cleaning and missing value processing, the data is standardized. Because the dimensions and ranges of different features are different, in order to prevent some features from having too great influence on the results in model training, Min-Max Normalization method is adopted to transform all eigenvalues into the range of [0,1]. At the same time, for the characteristics of normal distribution, Z-score standardization method is also used to ensure the uniformity of data and the effectiveness of analysis.

Through the above steps, it has laid a solid foundation for the subsequent data mining. In addition, in the application of Transformer architecture, a multi-layer encoder-decoder model based on self-attention mechanism is constructed to process the sequence data in carbon emission data. The parameter setting of this model includes the number of self-attention layers is 6, and the learning rate is 0.001. Adam optimizer is used for training, which aims to capture the long-range dependence in data and enhance the recognition ability of complex data patterns. Through the application of these algorithms, the hidden information in carbon emission data is successfully mined, which provides valuable knowledge support for power enterprises. In addition, based on the DSR model proposed by the United Nations Commission on Sustainable Development and the results of machine learning algorithm, this study constructs a carbon audit evaluation system consisting of 18 indicators to effectively evaluate and optimize the carbon emission management of the power industry. In the process of data analysis, regression analysis and cluster analysis are used to ensure the scientificity and integrity of the evaluation system. In the regression analysis, multiple linear regression and LASSO regression (minimum absolute contraction and selection operator) are used to establish the relationship between carbon emission data and various evaluation indexes, and the value of regularization parameter λ is set to 0.1 to balance the model complexity and prediction accuracy. By analyzing the coefficients of the regression model, the key factors affecting carbon emissions are determined, which provides targeted suggestions for enterprises’ carbon emission reduction strategies. In the aspect of clustering analysis, the carbon emission data of power enterprises are classified by K-means algorithm, and the number of clusters K is set to 3, and the best number of clusters is selected by elbow rule and contour coefficient analysis. This analysis makes it possible to identify enterprises that have outstanding performance in carbon emission reduction and those that need improvement, thus providing a basis for further carbon management strategy optimization.

There are significant differences in energy structure, economic development level and environmental policies in different regions, which challenges the effectiveness of carbon footprint management model. In some developing countries, such as South Africa and India, fossil fuels are still the main energy source. Therefore, carbon emission control strategies should focus on improving the efficiency of fossil fuels and promoting clean energy substitution. In contrast, in Europe and other places, with the progress of renewable energy technology, the policy focus has shifted to promoting the large-scale application of renewable energy. Therefore, the proposed carbon footprint management model has certain flexibility, and can adapt to the specific conditions of different regions by adjusting the input parameters to achieve more accurate carbon emission prediction and management. When building a carbon footprint calculation model based on big data and AI technology, this study pays special attention to collecting and processing a variety of key data, including but not limited to fuel types (such as coal, natural gas, nuclear energy and renewable energy), power generation, power generation efficiency, equipment operation status and geographical location. The accurate acquisition and processing of these data is the basis to ensure the accuracy and reliability of the model. For coal-fired power plants, the proportion of different quality coal and its corresponding carbon emission coefficient are recorded in detail. For wind power station, the influence of wind speed change on power generation efficiency is considered.

## Results and discussion

### Raw data standardization processing

**Fig 5:**
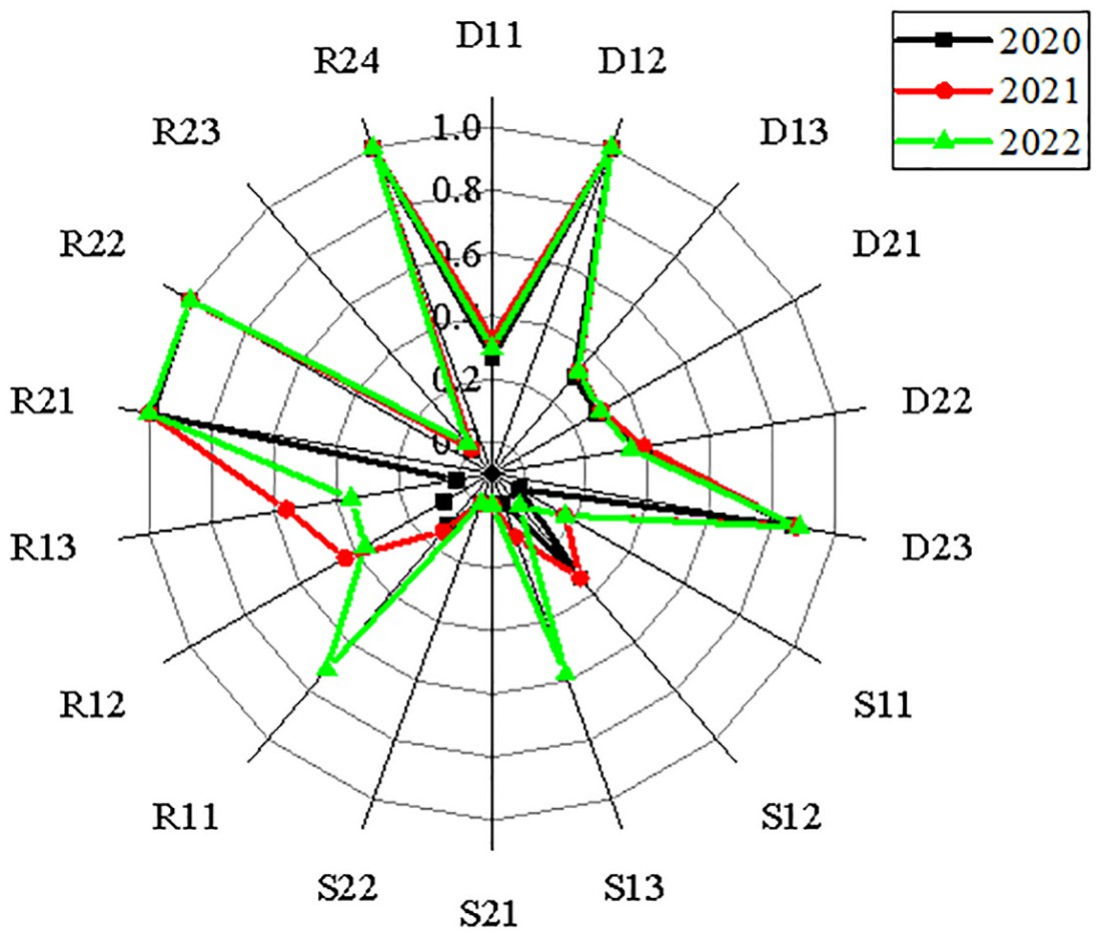
Data standardization results of China Resources Power enterprise.

**Fig 6:**
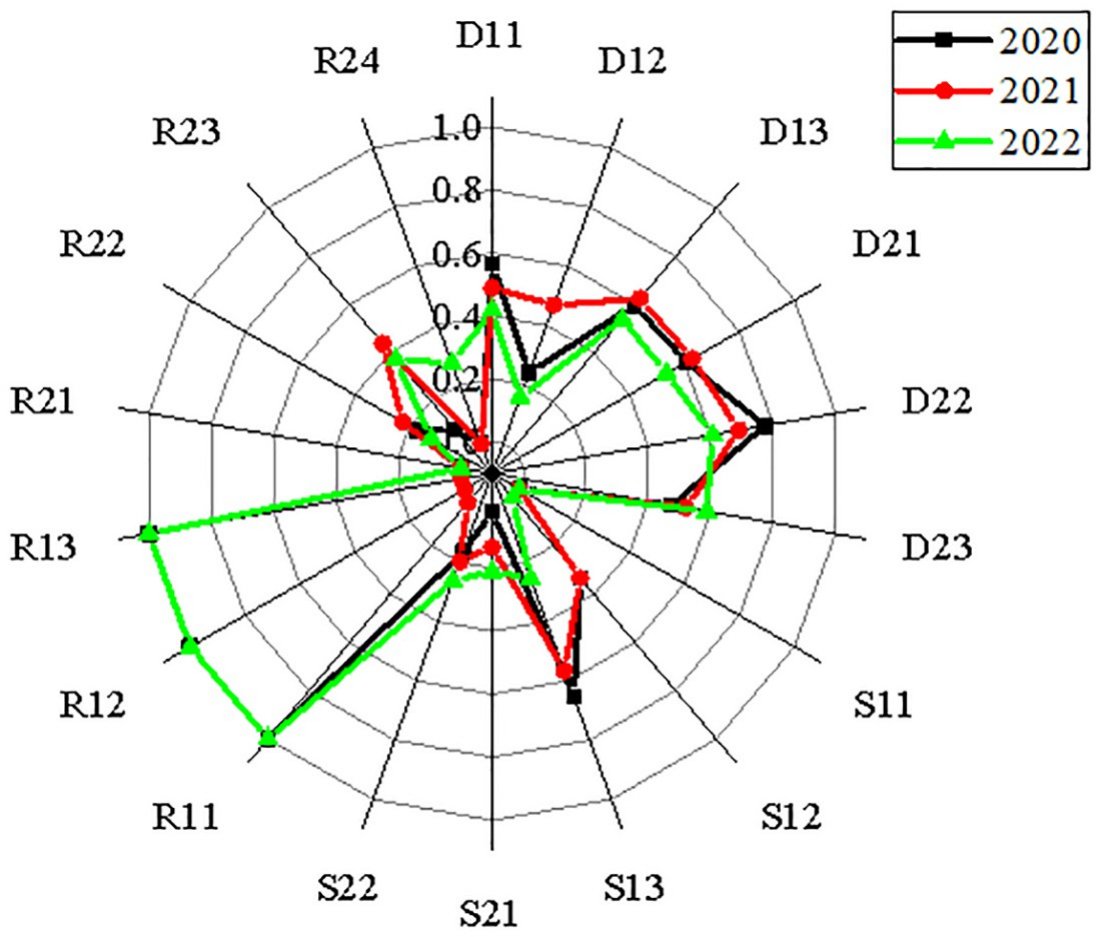
Data standardization results of Datang International Power Generation Co., Ltd.

This study uses the carbon audit evaluation index system of the power industry, constructed in Table 4, along with the comprehensive evaluation method of entropy weight—TOPSIS to explore measures to ensure the effective application of the carbon audit evaluation index system. The study uses two power companies, China Resources Power and Datang International Power Generation Co., Ltd., as examples. It analyzes the effectiveness of carbon reduction and emission reduction in detail. Through the flush database and the official website of each enterprise, the original data from 2020 to 2022 needed to build the index system are obtained. Because each evaluation index’s nature and unit may vary, dimensionless original data must be used while building the carbon audit assessment index system for the power sector. Figs 5 and 6 display the two enterprises’ standardized matrices.

### Comprehensive evaluation of carbon governance in electric power enterprises

**Fig 7:**
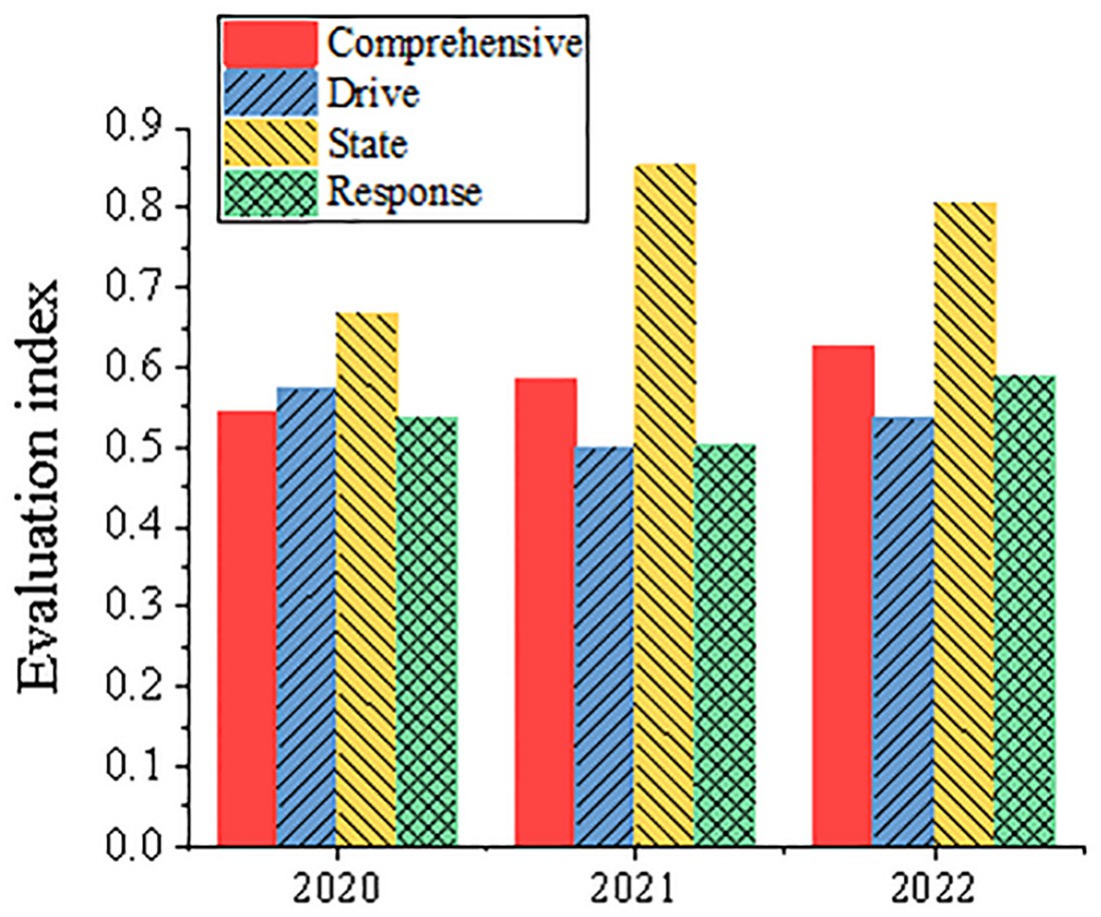
Index statistical results of comprehensive evaluation of China Resources Power and evaluation of various dimensions.

**Fig 8:**
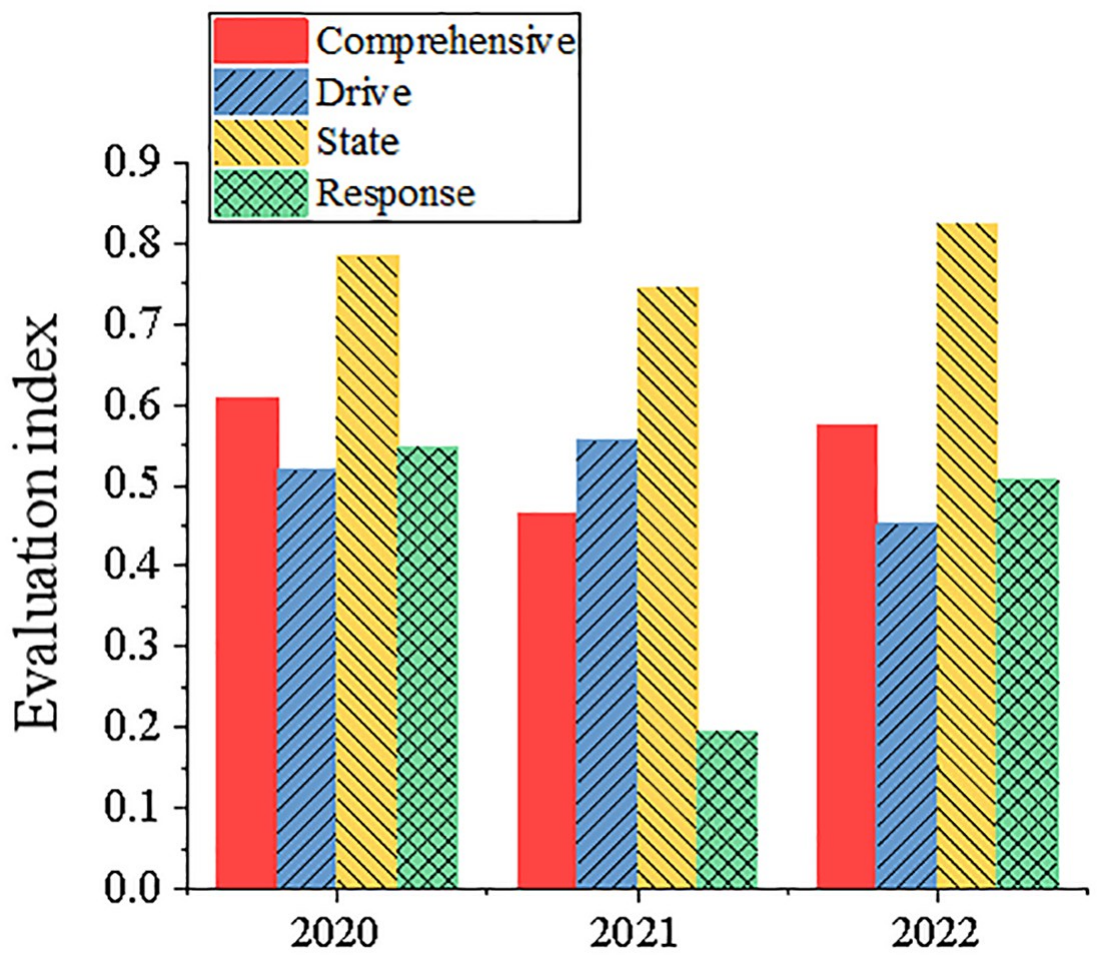
Index statistical results of Datang International Power Generation Co., Ltd. comprehensive evaluation and various dimensions evaluation.

Figs 7 and 8 show the comprehensive evaluation results of China Resources Power and Datang International Power Generation Co., Ltd. from 2020 to 2022, and the index statistical results of each dimension evaluation. It shows that the comprehensive evaluation index of China Resources Power rose from 0.5458 in 2020 to 0.627 in 2022. This is mainly due to a series of positive measures taken by the company in clean energy investment, such as vigorously developing hydropower, wind power and photovoltaic projects, among which the construction of Jiangsu Lianshui wind farm has significantly reduced carbon emissions. China Resources Power gradually reduced the proportion of thermal power generation by vigorously promoting clean energy, such as hydropower, wind power and photovoltaic, thus significantly reducing carbon emissions. For example, Jiangsu Lianshui Wind Farm built by this enterprise has an annual power generation of 1.19 billion kWh, which can replace standard coal consumption by 11,700 tons and reduce carbon dioxide emissions by 26,000 tons. In addition, China Resources Power also actively invests in new energy projects, such as Chibi million-kilowatt new energy base, fishery and light complementary power generation project, etc. These projects not only provide a lot of green energy, but also significantly reduce carbon dioxide emissions. The comprehensive evaluation index of Datang International Power Generation Co., Ltd. is fluctuating, and it dropped to 0.4658 in 2021. It reflects the challenges it faces in carbon emission reduction. Especially in the response dimension, the company’s poor performance in sulfur dioxide (SO_2_) and carbon dioxide (CO_2_) emission reduction led to a significant decline in the evaluation index. This phenomenon can be partly attributed to changes in the external policy environment, such as the adjustment of emission reduction policies in some regions and the impact of power market fluctuations on the carbon emission costs of enterprises. In addition, the management strategies of enterprises in equipment maintenance and investment in environmental protection facilities may also be insufficient, resulting in poor performance in emission reduction. State dimension indicators have performed well in recent years, and the evaluation indexes have all reached above 0.7. In the response dimension, enterprises have encountered significant challenges. Especially in 2021, the evaluation index of this dimension dropped to 0.1955, when analyzing the potential influencing factors, the successful experience of China China Resources Power mainly benefits from its large-scale investment in clean energy projects, such as Lianshui wind farm built in Jiangsu and the million-kilowatt new energy base in Chibi, Hubei. These projects not only provide a lot of green energy for the company, but also significantly reduce coal consumption and carbon dioxide emissions. However, the volatility performance of Datang International Power Generation Co., Ltd. reflects its lack of flexibility and adaptability in response to market and policy changes, especially in 2021, its strategy adjustment in SO_2_ and CO_2_ emission reduction is not in place. After in-depth analysis of the reasons, it is considered that China Electric Power Resources Company can effectively reduce carbon emissions while the evaluation index is rising, mainly because of its strategic investment in clean energy, which gradually reduces the proportion of thermal power and increases the proportion of wind power and photovoltaic power generation, thus effectively reducing carbon emissions. In contrast, the progress of Datang International Power Generation Co., Ltd. is relatively slow, and the implementation effect of its carbon emission reduction measures failed to meet expectations, which may be due to its dependence on existing technologies and its untimely response to environmental policies. Based on the above analysis results, specific management suggestions are put forward for the two companies. For China Electric Power Resources Company, it is suggested to continue to strengthen investment in clean energy projects, further optimize the existing energy structure, and improve the real-time and accuracy of carbon emission monitoring to ensure the effectiveness of emission reduction measures. For Datang International Power Generation Co., Ltd., people should focus on improving the investment in environmental protection technology and strengthening the rapid response to policy changes, especially in the field of sulfur dioxide and carbon dioxide emission reduction technology. It is suggested to speed up the technology update and management optimization to ensure that the expected emission reduction targets can be achieved.

Table 5 shows the data comparison results before and after the improvement of carbon audit evaluation system of power enterprises in China and other countries:

**Table 5:** Data comparison results before and after the improvement of carbon audit evaluation system of power enterprises in China and other countries.

Index	Before improvement (China)	After improvement (China)	Before improvement (other countries)	After improvement (other countries)
Total carbon emissions (ten thousand tons)	5000	4000	4800	3600
Accuracy of carbon audit (%)	70	85	75	90
Investment in low-carbon technology (100 million yuan)	20	50	25	55
Carbon emission intensity (ton/GWh)	0.8	0.6	0.75	0.55
Proportion of clean energy (%)	25	35	30	40

Table 6 shows the changes of carbon audit evaluation indexes of China’s electric power enterprises (China Electric Power Company and Datang International Electric Power Company):

**Table 6:** Changes of carbon audit evaluation indexes of China’s electric power enterprises (China Electric Power Company and Datang International Electric Power Company).

Index	China Electric Power Company (2020)	China Electric Power Company (2022)	Datang International Electric Power Company (2020)	Datang International Electric Power Company (2022)
Overall evaluation index	0.5458	0.627	0.5237	0.4958
Carbon emission reduction (ten thousand tons)	200	350	180	150
Installed capacity of clean energy (10,000 kilowatts)	5000	7000	4500	4600
SO2 emission reduction (ten thousand tons)	50	70	40	35
CO2 emission reduction (ten thousand tons)	300	400	250	200
Accuracy of carbon audit (%)	72	85	68	75

Under the background that the current carbon audit evaluation system and standards are not perfect, it is suggested to further improve these systems and standards in combination with the actual situation and advanced experience at home and abroad. By analyzing the data in Tables 5 and 6, the improvement of the system and standards has significantly improved the performance and effect of carbon audit in China’s power enterprises. According to the data in Table 5, before the improvement, the total carbon emission of China’s power enterprises is 50 million tons, the accuracy of carbon audit is 70%, the investment in low-carbon technology is 2 billion yuan, the carbon emission intensity is 0.8 tons/GWh, and the proportion of clean energy is 25%. After the improvement, these indicators have been significantly improved. The total carbon emission decreased to 40 million tons, the accuracy of carbon audit increases to 85%, the investment in low-carbon technology increases to 5 billion yuan, the carbon emission intensity decreases to 0.6 tons/gwh, and the proportion of clean energy increases to 35%. These data show that the improved carbon audit evaluation system has made remarkable progress in carbon emission control and low-carbon technology investment in China’s power enterprises, which reflects the effectiveness of the system improvement. Further analysis of the data in Table 6 shows the changes of carbon audit evaluation indexes of China Electric Power Company and Datang International Electric Power Company between 2020 and 2022. The overall evaluation index of China Electric Power has increased from 0.5458 in 2020 to 0.627 in 2022, the carbon emission reduction has increased from 2 million tons to 3.5 million tons, and the installed capacity of clean energy has increased from 50 million kilowatts to 70 million kilowatts. These data show that after the implementation of improvement measures, China Electric Power Company has significantly improved the accuracy and effect of carbon audit, and made remarkable progress in carbon emission reduction and clean energy investment. In contrast, the overall evaluation index of Datang International Power Company has decreased from 0.5237 to 0.4958. Although the accuracy of its carbon audit has also improved, the reduction of carbon emissions and the installed capacity of clean energy have changed little. This situation shows that although Datang International has improved the accuracy of carbon audit, it faces certain challenges in actual carbon emission reduction and clean energy investment. These data support the conclusion that there is still room for improvement in the current carbon audit evaluation system and standards.

By further optimizing the carbon audit system and combining the advanced experience in China and other countries, people can better meet the actual needs of power enterprises and improve the accuracy and effectiveness of carbon audit. The improved system can not only evaluate carbon emissions more accurately, but also promote enterprises to make greater progress in low-carbon technology investment and clean energy transformation, thus promoting the green development of the whole industry. Specifically, firstly, it is necessary to deeply analyze the operating characteristics of power enterprises and the specific links of carbon emissions, and clarify the key indicators of carbon audit evaluation. These indicators should cover all aspects of power production, transmission and distribution, consumption and so on to ensure that the evaluation system can fully capture the carbon emissions of enterprises. For example, indicators such as carbon emission intensity per unit power generation, clean energy ratio, carbon capture and storage capacity can be considered. Secondly, it draws on advanced theoretical frameworks such as the DSR model to construct a structured carbon audit evaluation system. DSR model provides a systematic analysis framework for carbon emission management by identifying drivers, evaluating current state and analyzing response measures. In the power industry, the drivers may include energy structure, technological progress and policy environment. The state involves carbon emissions, energy efficiency and other indicators. The response measures include the development of clean energy and the application of energy saving and emission reduction technologies. Furthermore, people should make full use of international authoritative data sources such as IEA CO₂ Emissions Database and refer to their carbon emission factors and accounting methods to formulate unified and standardized carbon footprint accounting standards. This not only helps to improve the accuracy of carbon audit, but also enhances the comparability of carbon emission data between different enterprises. In addition, a set of transparent reporting guidelines should be established to clarify the collection, calculation and reporting process of carbon emission data, as well as the third-party audit and verification mechanism. This will help to improve the transparency and credibility of carbon audit and promote the trust among enterprises, government and the public. Finally, the improvement of the evaluation system and standards should be a dynamic process, which needs to be updated and adjusted regularly according to the development trend of the industry, technological progress and policy changes. At the same time, experts and scholars, business representatives and policy makers inside and outside the industry are encouraged to participate in the construction and improvement of the evaluation system to ensure that the evaluation system is scientific, practical and forward-looking.

## Conclusions

In light of the dire consequences of climate change, power enterprises’ carbon footprint management is critical to reaching the “double carbon” objective since it is one of the primary areas of energy consumption and carbon emissions. Based on the carbon footprint management of power enterprises, this study constructs a scientific and systematic carbon footprint management index system by integrating big data, AI and advanced carbon audit evaluation model, aiming at providing strong support for power enterprises to formulate more accurate and effective carbon emission reduction strategies. The carbon footprint calculation model of power enterprises is effectively created using AI and big data technologies. The model can accurately collect and calculate the carbon emission data of the whole chain and multi-dimensions of power enterprises, which lays a solid foundation for further in-depth analysis and strategy formulation. Through this model, people can not only clearly grasp the total amount and structure of carbon emissions of power enterprises, but also deeply analyze the contribution of different links and factors to carbon emissions. It offers a solid scientific foundation for developing emission reduction plans. The carbon emission data of power enterprises is thoroughly mined and analyzed using a machine learning algorithm, which effectively identifies the important factors influencing carbon emission and the potential for emission reduction. With the introduction of the DSR model, the industry’s reaction strategies to reduce carbon emissions, as well as the factors driving carbon emissions, can all be completely reflected. This offers a more thorough and in-depth perspective for evaluating carbon audits.

In this study, a carbon footprint management model of power enterprises based on AI is proposed, and it is analyzed with the data of IEA CO_2_ emission database. Compared with the existing literature, this study has some innovative contributions. Firstly, AI technology is introduced into the carbon footprint management model for the first time, and an accurate carbon footprint calculation model is established through big data and machine learning algorithm. This model not only improves the accuracy of carbon footprint calculation, but also effectively excavates the key factors affecting carbon emissions, which provides scientific decision support for power enterprises to reduce emissions. Secondly, based on the DSR model, a comprehensive carbon audit evaluation index system is constructed, which can systematically evaluate the performance of power enterprises in carbon emission reduction and reveal the existing problems and shortcomings. This evaluation system fills the shortcomings of the current carbon audit evaluation standards and provides new ideas and methods for future carbon footprint management. In addition, in the process of data analysis, entropy weight method and TOPSIS comprehensive evaluation method are comprehensively applied, which makes the results of carbon audit evaluation more objective and reliable. The effectiveness of this method in carbon footprint management is successfully proved by verifying the actual data of China electric power enterprises. The results of this study have important practical application value for carbon footprint management of power enterprises. Firstly, through the carbon footprint management model, power companies can accurately evaluate their carbon emission levels, and formulate scientific emission reduction strategies according to the results provided by the model to identify the main carbon emission sources and take effective measures to reduce carbon emissions to achieve green production and sustainable development. Secondly, the proposed carbon audit evaluation system based on DSR model provides a systematic carbon emission evaluation tool for power enterprises, enabling them to conduct carbon audits regularly, find and solve problems in the process of carbon emission reduction in time, and improve the transparency and accuracy of carbon management. In addition, the model and evaluation system in this study not only have a guiding role for enterprises, but also provide data support for government departments to formulate carbon emission policies, help them evaluate the implementation effect of policies more accurately, and adjust and optimize relevant policies accordingly. Although this study mainly focuses on the carbon footprint management of electric power enterprises in China. An efficient and accurate carbon footprint calculation model is constructed by introducing AI technology and machine learning algorithm. It is realized that this model may have some limitations in adapting to the energy structure and regulatory environment of different countries and regions. There are significant differences in energy consumption patterns, technical levels, economic conditions and environmental policies among countries, which may affect the applicability and forecasting accuracy of the model. Some countries may be more dependent on fossil fuels, while others may have a higher proportion of renewable energy. In addition, different regulatory frameworks also have an important impact on carbon emission management. Therefore, when the proposed carbon footprint management model is applied to other countries and regions, it needs to be adjusted and optimized according to the local specific conditions. This may involve the selection of model input parameters, the modification of weight distribution methods and the re-identification of carbon emission drivers in specific situations. Nevertheless, this study provides a flexible and extensible basic framework, which provides a useful reference for future carbon footprint management research in different backgrounds.

Although some achievements have been made, there are still some shortcomings. Firstly, the existing carbon footprint management models are mostly based on traditional statistical methods and empirical equations, and it is difficult to comprehensively and accurately reflect the carbon emissions of power enterprises. Secondly, the application of AI in carbon footprint management is still in its infancy, and a systematic solution and a mature application model have not yet been formed. In addition, the current research lacks a carbon footprint management model and emission reduction strategy for the characteristics of power enterprises, which is difficult to meet the actual needs of low-carbon transformation in the power industry. In view of these shortcomings, the future research can be prospected from the following aspects. First, it can further optimize and improve the carbon footprint management model, and develop more accurate and scientific carbon emission calculation methods in combination with the specific characteristics of power enterprises. In order to increase the precision of carbon emission forecast and analysis, the second is to encourage the extensive use of AI technology in carbon footprint management. This involves investigating more effective data processing and pattern recognition algorithms. Through these efforts, it is expected to provide more powerful support and guidance for the low-carbon transformation of power enterprises and even the whole society. Future research can further optimize the carbon audit evaluation system, combine more advanced theoretical framework and actual situation, and formulate more accurate carbon footprint management standards. At the same time, attention should be paid to the introduction of international standards to improve the accuracy and comparability of carbon emission data. In addition, future research can also explore how to use AI technology to further improve the accuracy and efficiency of carbon emission management.

## Supporting information

S1 Data (XLS)
